# Role of lateral amygdala calstabin2 in regulation of fear memory

**DOI:** 10.1186/s13041-020-00576-7

**Published:** 2020-03-09

**Authors:** Ren-wen Han, Zhi-peng Liu, Hong-ru Lin, Ao-wen Tian, Yun-fei Xiao, Jie Wei, Ke-yu Deng, Bing-xing Pan, Hong-bo Xin

**Affiliations:** 1 The National Engineering Research Center for Bioengineering Drugs and the Technologies, Institute of Translational Medicine, Nanchang University, Nanchang, China; 2 Laboratory of Fear and Anxiety Disorders, Institute of Life Science, Nanchang University, Nanchang, China; 3 Department of Physiology, Medical College of Nanchang University, Nanchang, China; 4 College of Life Sciences, Nanchang University, Nanchang, China

**Keywords:** calstabin2, Ryanodine receptor, Lateral amygdala, Fear memory, Long-term potentiation

## Abstract

Calstabin2, also named FK506 binding protein 12.6 (FKBP12.6), is a subunit of ryanodine receptor subtype 2 (RyR2) macromolecular complex, an intracellular calcium channel. Studies from our and other’s lab have shown that hippocampal calstabin2 regulates spatial memory. Calstabin2 and RyR2 are widely distributed in the brain, including the amygdala, a key brain area involved in the regulation of emotion including fear. Little is known about the role of calstabin2 in fear memory. Here, we found that genetic deletion of calstabin2 impaired long-term memory in cued fear conditioning test. Knockdown calstabin2 in the lateral amygdala (LA) by viral vector also impaired long-term cued fear memory expression. Furthermore, calstabin2 knockout reduced long-term potentiation (LTP) at both cortical and thalamic inputs to the LA. In conclusion, our present data indicate that calstabin2 in the LA plays a crucial role in the regulating of emotional memory.

## Introduction

Endoplasmic reticulum (ER) extends throughout the neurons, including the soma, arborized dendrites, dendritic spines, axons, and axon terminals. Therefore ER supports functionally diverse roles [1]. ER Ca^2+^ release occurs via ryanodine receptors (RyRs) and inositol (1,4,5)-trisposphate receptors (IP3Rs), and is involved in modulating neurotransmitter release, gene transcription and synaptic plasticity [1].

Three isoforms of RyRs (RyR1, RyR2 and RyR3) are all distributed in the brain [2]. Among them, RyR2 was demonstrated to be involved in modulating spatial memory, as indicated by 1) hippocampal RyR2 expression was increased after Morris water maze task [3, 4], and 2) lateral ventricle injection of RyR2 antisense oligonucleotides weakened memory in the mouse passive avoidance test [5].

Calstabin2, also known as FK506 binding protein 12.6 (FKBP12.6, also named FKBP1b), is a critical regulatory subunit of the RyR2 macromolecular complex. Liu et al. reported that chronic stress resulted in depletion of the calstabin2 from RyR2 and intracellular calcium leak, therefore impaired spatial memory and long-term potentiation (LTP) at the hippocampal CA3-CA1 connection [6]. Recently, our group has shown that genetic deletion of calstabin2 impaired spatial memory, induced RyR2 leak, and reduced LTP at the hippocampal CA3-CA1 connection. These results indicate hippocampal calstabin2 might regulate spatial memory by modulating ER Ca^2+^ release via RyR2. In addition, calstabin2 and RyR2 were demonstrated to be involved in the aging-related hippocampal neuronal Ca^2+^ dysregulation and spatial memory deficiency [7–9]. In murine of Alzheimer disease (AD) models, dissociation of calstabin2 from the RyR2 complex is also responsible for the cognitive dysfunction [10].

The amygdala is critical for threat memory formation [11]. RyR2 is highly distributed throughout the brain, including amygdala [2]. Calstabin2 is also abundantly expressed in the brain [12], and is detected in the amygdala in our present experiment. So far, whether calstabin2 could regulate fear memory in the lateral amygdala (LA) was not yet known, and we attempted to address this issue in the present study.

## Methods and materials

### Animals

Calstabin2 KO mice of C57BL/6 background were generated using homologous recombination to disrupt exon 3 of the calstabin2 gene, as previously described [13]. Animals were housed in an animal room that was maintained at 22 ± 2 °C with a 12-h light: 12-h dark cycle. Food and water were available ad libitum. All the experimental procedures were approved by the Ethics Committee of Nanchang University and performed in accordance to the guidelines of Animal Use and Care of NIH and the ARRIVE, and the results were reported in line with these guidelines.

### Adeno-associated virus (AAV)

AAV-pAKD-CMV-bGlobin-eGFP-H1-shRNA-NC (AAV2/8) and AAV-pAKD-CMV-bGlobin-eGFP-H1-shRNA (AAV2/8) were purchased from Obio Biotechnology Co., Ltd. The titer of both AAVs was quantified to be 1.2 × 10^13^ viral genomes/ml. The target sequence of the control shRNA (shRNA-NC) was TTCTCCGAACGTGTCACGT; and the target sequence of the calstabin2 shRNA was GCACTACACAGGAATGCTT.

### Surgery and drug infusion

Mice at age of 5–6 weeks were anesthetized with sodium pentobarbital (70 mg/kg, Sigma) and placed in a stereotaxic frame (RWD Life Science Co. Ltd., China). A glass micropipette with a tip diameter of approximately 30 μm was connected to a 10 μl Hamilton syringes mounted on a microdrive pump (RWD, China). The micropipette filled with AAV solution was inserted to the LA (1.8 mm posterior to bregma, ±3.4 mm lateral to midline, 4.2 mm ventral to bregma). Then 0.5 μl solution was infused over a period of 10 min. After the cessation of infusion, the cannula remained in place for 5 min to allow for drug diffusion. Three weeks after surgery, mice were used for behavioral test.

### Histology

After behavioral test, mice were sacrificed by decapitation and whole brains were removed and fixed in 4% paraformaldehyde overnight at 4 °C. Coronal sections (50 μm) were cut with a vibratome (VT 1000S, Leica Microsystems). The virus expression was verified with a fluorescence microscope (BX63, Olympus). Only mice that showed highly specific expression in the LA were included in data analysis.

### Fear conditioning

The tests were performed on male littermates at 8–9 weeks of age as our previous report [14]. The experimenter was blind to the genotypes or drug treatments. The conditioned stimulus (CS) was a 10 s, 3 kHz tone with an intensity of 80 dB. The unconditioned stimulus (US) was a 1 s, 0.5 mA electric shock co-terminated with the CS. On the first day of the experiment, the mouse was placed in the training chamber (Med Associates, St. Albans, VT**)** with a metal grid floor for shock application for 5-min habituation. During the habituation, 5 CS were presented without US at variable intervals of 30–60 s. On the second day, the mouse was put back to the chamber and 2 min later, 5 CS-US pairing which co-terminated were presented in variable intervals (60–180 s). The mice remained in the context for an additional 30 s before returning to their home cages. After each trial, the chamber was cleaned with 70% EtOH. 24 h after the training, the cued conditioning was assessed in a different context; the CS with 1 min duration was given after a 1 min habituation period. Freezing was defined as a complete lack of movement measured by a video-based system.

### Western blot

The mice were anesthetized with isoflurane and 300-μm thick coronal brain sections including the LA were prepared using a vibratome. The LA tissue samples were obtained by dissecting the LA from the brain sections according to the mouse brain anatomy under stereoscopic microscope (Leica). For AAV infusion mice, only areas with fluorescence signal were dissected. According to the previous report [6], the dissected LA tissues were homogenized in ice-cold buffer (250 mM sucrose, 5 mM HEPES-KOH, pH 7.4, 1 mM EGTA, and protease inhibitors). Then the tissue homogenate was centrifuged at 1000 g for 10 min, and the supernatant was then collected and centrifuged at 8000 g for 10 min. The resulting supernatant was collected and centrifuged at 40,000 g for 45 min. After centrifugation, the resulting pellet was resuspended in resuspension buffer (250 mM sucrose, 5 mM HEPES-KOH, pH 7.4, and protease inhibitors) and gently homogenized. The proteins were separated by electrophoresis on 15% SDS-PAGE gel and transferred to PVDF membrane. The membrane was blocked with 5% non-fat milk for 1 h and incubated at 4 °C overnight with the primary antibodies (calstabin2: OmnimAbs, OM253226, 1:1000; TBP: Abcam, ab818, 1:1000), and then incubated with appropriate secondary antibodies (Thermo fisher, #31430 for mouse, #31460 for rabbit, dilution: 1:5000;) at room temperature for 1 h. Images were obtained by the Bio-Rad Molecular Imager Chemi Doc XR+ System with Image Lab Software. Expression of calstabin2 was normalized with the reference TBP.

### Electrophysiology

Amygdala slices were prepared according to the previous experimental protocol [15]. Briefly, mice were anesthetized with diethyl ether and decapitated, whereupon brains were quickly immersed in ice-cold oxygenated (95% O_2_/5% CO_2_) sucrose physiological extracellular solution containing (in mM): 80 NaCl, 3.5 KCl, 4.5 MgSO_4_, 0.5 CaCl_2_, 1.25 NaH_2_PO_4_, 90 sucrose, 10 glucose, and 22 NaHCO_3_ (pH 7.30). Coronal slices (320 μm) containing the LA were cut in ice-cold sucrose solution using a vibratome (VT 1000S, Leica Microsystems) and incubated in a maintenance chamber filled with artificial cerebrospinal fluid (ACSF) at 34 °C. After 30 min recovery time, slices were transferred to room temperature for at least 1 h before recordings.

LTP experiments were performed at 29 ± 1 °C in a submersion-type recording chamber perfused at ~ 2 ml/min with ACSF. Recording electrodes were pulled from thick-wall borosilicate glass tubes by using a horizontal pipette puller (P97; Sutter Instrument Co., Novato, CA). Patching pipette resistance was 4–6 MΩ when filled with intracellular solution containing (in mM): 130 K-Gluconate, 5 NaCl, 1 MgCl_2_, 0.2 EGTA, 10 HEPES, 2 Mg-ATP, 0.1 Na-GTP, 1000 sucrose. Series resistance (Rs) was in the range of 10–20 MΩ, and the data were not included in analysis when Rs changed more than 20% during recording. To obtain evoked synaptic responses, the stimulation electrode was placed in the external capsule and intermediate capsule to record input information from the cortex and thalamus, respectively. In wild-type and calstabin2 KO mice, stimulus intensities were adjusted to evoke EPSC amplitudes of 100–250 pA in the LA primary neurons. EPSCs were monitored every 20 s, and interleaved test pulses were used to monitor the recording quality (series resistance, pipette capacitance compensation). For LTP induction, the neuronal membrane potential was held at + 30 mV and the pairing protocol included 100 presynaptic stimuli at 2 Hz delivered to either cortical or thalamic pathway. Stimuli intensity was adjusted to produce synaptic responses with an amplitude that was ~ 30% of maximum. LTP was quantified by normalizing the data collected in 30–40 min after LTP induction to the mean value of the amplitude of 10 min baseline before LTP induction.

### Statistical analysis

Statistical analysis was conducted using SPSS 17.0. Independent Student’s *t*-test, one-way followed by Bonferroni*-*test and ANOVA with repeated measures were used. The specific tests used for each experiment are provided in the respective figure legend. *P* < 0.05 was considered significant. Data were expressed as mean ± SEM.

## Results

### Impairment of cued fear memory in calstabin2 KO mice

**Fig. 1:**
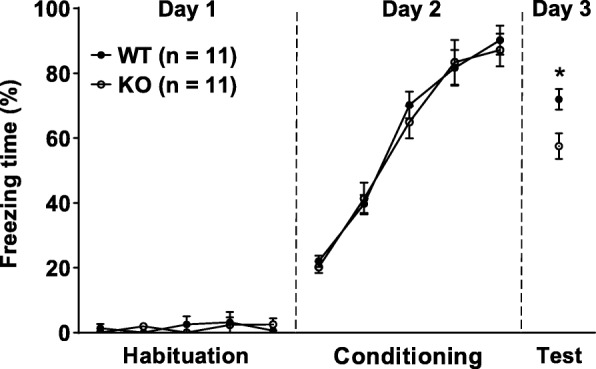
Genetic knockout (KO) of calstabin2 impairs long-term cued fear memory. Calstabin2 KO did not affect freezing response during the habituation (day 1: *F*_1, 20_ = 0.339, *p* = 0.567, ANOVA with repeated measures) and conditioning phases (day 2: *F*_1, 2_ = 0.038, *p* = 0.847, ANOVA with repeated measures) compared with the wildtype (WT) mice. During the test phase, KO mice froze less often to conditioned stimulus than WT ones did (day 3: *p < 0.05, independent student’s *t* test)

Before conditioning, both WT and calstabin2 KO mice showed similar response to the presentation of CS during the habituation and conditioning phases (Fig. 1). When placed in the testing context 24 h after training, the KO mice had shorter freezing time to CS than their WT counterparts (*p* < 0.05; Fig. 1), indicating that calstabin2 deletion causes impairment in long-term cued fear memory.

### Impairment of cued fear memory in LA calstabin2 knockdown mice

**Fig. 2:**
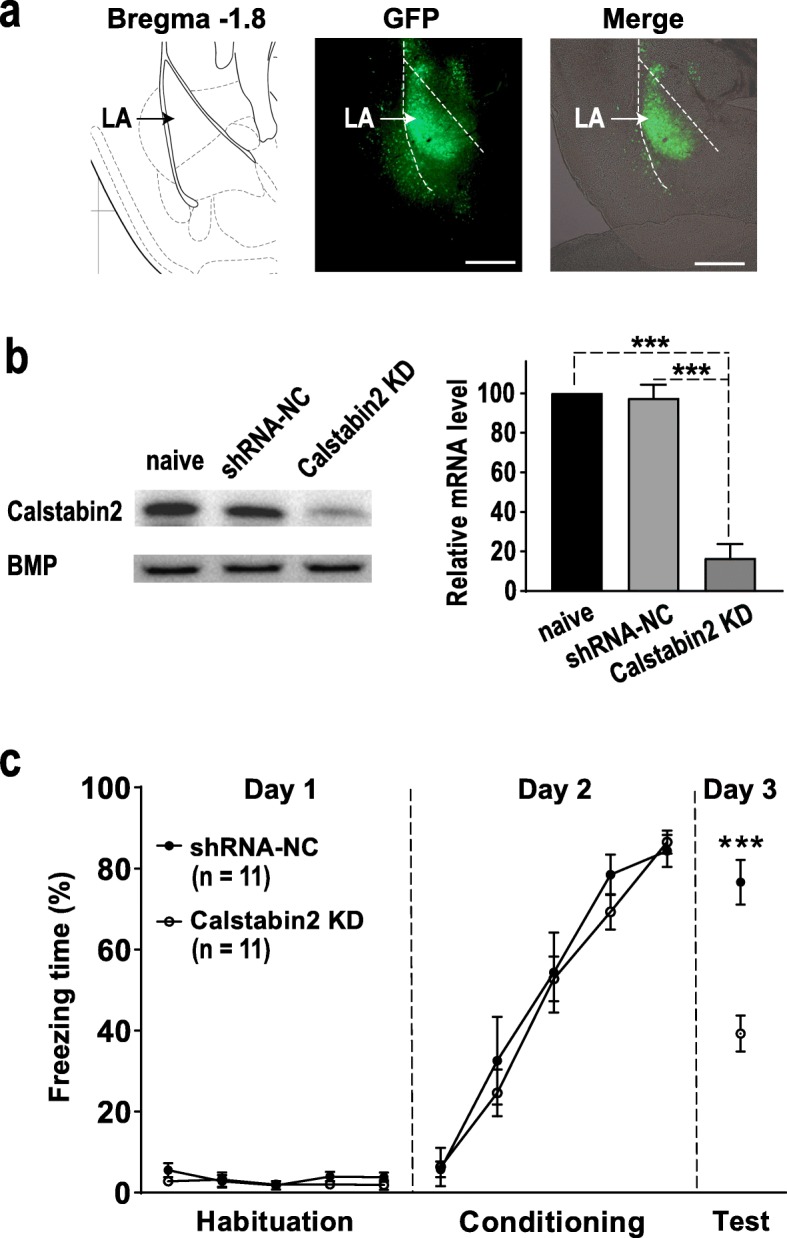
Lateral amygdala (LA) calstabin2 knockdown (KD) impairs long-term cued fear memory. **a** Representative AAV-infected neurons (green) in the LA. Scale bar: 200 μm. **b** Western blot showing calstabin2 protein levels in the LA of naive mice, shRNA-NC infected mice, and calstabin2 KD mice. Left: representative Western blot. Right: calstabin2 expression histograms. The *y*-axis indicates the calstabin2 protein expression level for each condition relative to naive control. The calstabin2 protein level for each condition was normalized to the BMP level. *N* = 3 for each group. Statistics, One-way ANOVA and Bonferroni post-hoc test. ***p < 0.001 compared with naive and shRNA-NC groups. **c** LA calstabin2 KD did not affect freezing response during the habituation (day 1: *F*_1, 20_ = 1.125, *p* = 0.301, ANOVA with repeated measures) and conditioning phases (day 2: *F*_1, 20_ = 0.378, *p* = 0.545, ANOVA with repeated measures). During the test phase, calstabin2 KD mice froze less often to conditioned stimulus than shRNA-NC infected ones did (day 3: ***p < 0.001, independent student’s *t* test)

To further investigate whether calstabin2 in the LA could regulate cued fear memory, we used AAV-mediated delivery of calstabin2 shRNA to KD calstabin2 expression in LA neurons. The calstabin2 shRNA, but not the control shRNA infused into the bilateral LA robustly reduced calstabin2 protein level in the LA (*p* < 0.001; Fig. 2a and b). During the habituation and conditioning phases, calstabin2 KD and WT mice showed similar response to the CS (Fig. 2c). When animals were tested 24 h after the conditioning, the KD mice froze significantly less often to CS than the control mice did (*p* < 0.001), indicating that calstabin2 KD in the LA impairs long-term cued fear memory (Fig. 2c).

### Disruption of LTP induction in both cortical and thalamic inputs to the LA in calstabin2 KO mice

**Fig. 3:**
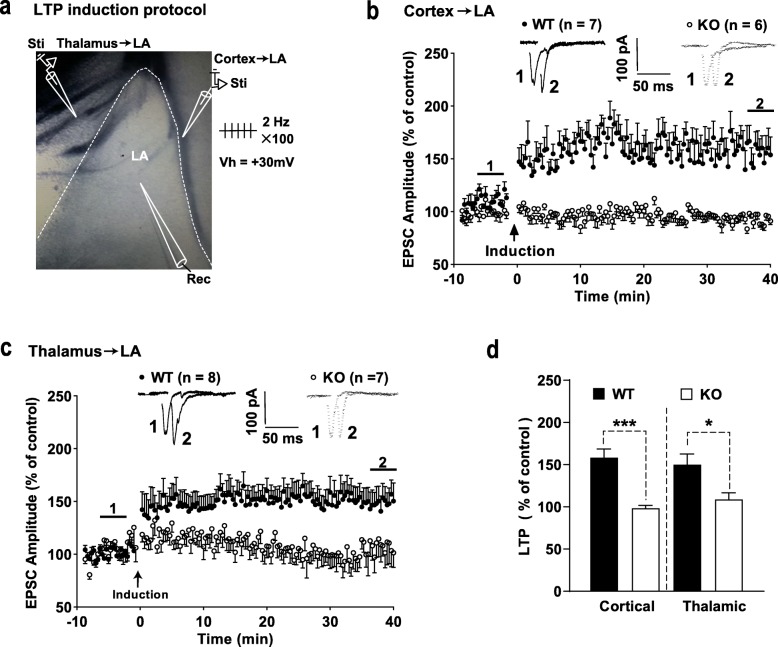
Genetic knockout (KO) calstabin2 disrupts long-term potentiation (LTP) induction both in cortical and thalamic inputs to the lateral amygdala (LA). **a** A schematic representation showing the experiment design in which 100 stimuli at 2 Hz were delivered to either cortical or thalamic afferents to the LA and LA neurons were held at + 30 mV. Sti and Rec indicate the location of stimulation and recording electrodes, respectively. **b, c** LTP in cortical (b) and thalamic (c) inputs to the LA from wild-type (WT) and KO mice. Insets represent excitatory postsynaptic currents (EPSCs) before (1) and after (2) LTP induction. **d** Summary plots of the data in **b** and **c**. Data were analyzed with independent Student’s *t*-test. *p < 0.05 and ***p < 0.001

LTP was induced by a pairing protocol (100 stimuli at 2 Hz delivered to presynaptic afferents with postsynaptic depolarization to + 30 mV) in cortical and thalamic inputs to the LA (Fig. 3a). LTP was readily evoked in WT mice in both afferents to the LA (Fig. 3b and c). Somewhat surprisingly, in calstabin2 KO mice, we failed to evoke LTP in both afferents (Fig. 3b and c). Comparison between genotypes showed that calstabin2 KO significantly suppressed LTP expression in both afferents (cortical: *p* < 0.001; thalamic: *p* < 0.05; Fig. 3d), suggesting that calstabin2 has a crucial role in regulating LTP induction in the LA.

## Discussion

Here, for the first time, we showed that long-term cued fear memory and LTP at cortical/thalamic afferents to the LA were impaired in calstabin2 KO mice. Moreover, calstabin2 KD in the LA also impaired long-term cued fear memory. Thus, our results suggest that LA calstabin2 plays a role in modulating emotional memory.

By using genetic KO mice, we have reported that deletion of calstabin2 impaired spatial memory in the Morris water maze and contextual fear conditioning tests, as well as decreased LTP at hippocampal CA3-CA1 connection [16]. Interestingly, Landfield and his colleagues found that the expression of hippocampal calstabin2 declined in aging rats and Alzheimer’s disease subjects [17]. Furthermore, they reported that disruption of calstabin2 induced Ca^2+^-dysregulation aging phenotype in young rat hippocampus, while overexpression of calstabin2 in the hippocampus reversed Ca^2+^ dysregulation, and rescued genomic regulation and cognitive impairment in aging rats [7–9]. In chronic stress and AD-like mice, the dissociation of calstabin2 from RyR2 resulted in the cognitive dysfunction, and were rescued by stabilizes calstabin2-RyR2 interaction [6, 10].. All these reports indicate involvement of hippocampal calstabin2-RyR2 system in spatial memory modulation.

RyR2 is widely distributed throughout the brain, including the amygdala, the key emotional memory regulation brain area, suggesting that calstabin2-RyR2 system may also take part in the modulation of fear memory [2]. As expected, the present data showed that disruption of calstabin2 gene resulted in impairment of long-term memory in the cued fear conditioning test. To further determine whether LA calstabin2 takes part in regulating of cued fear memory, LA calstabin2 is locally deleted by micro-infusion of AAV to the bilateral LA. We found that long-term cued fear memory expression was significantly impaired in the LA calstabin2 KD mice. During cued fear conditioning, signals generated by auditory CS enter the LA through projections from the auditory thalamus (input from the thalamus) and indirect projections from the auditory cortex (input from the cortex) [18]. LTP in both afferents is widely regarded as the synaptic mechanism underlying amygdala-related learning and memory [19]. Here, we found that, compared with WT mice, LTP was dramatically reduced both in both cortical and thalamic inputs to the LA. Thus, the present data showed LA calstabin2 could regulate cued fear memory.

Together, previous reports and our results suggest that calstabin2-RyR2 system takes part in modulating spatial and fear memories, which is consistent with their wide distribution throughout the brain. It is interesting to develop further study to understand whether this system regulates other forms of memories in other brain areas.

Both in chronic stressed and AD-like mice, calstabin2 was depleted from RyR2, resulting in intracellular calcium leak. Both pharmacological or genetic stabilizing RyR2-calstabin2 interaction could rescued spatial memory and hippocampal synaptic plasticity deficiency in these mice [6, 10]. These results indicate that calstabin2 could regulate synaptic plasticity and cognition via modulating ER Ca^2+^ release from RyR2 [20]. We showed that genetic disruption of calstabin2 in mice also resulted in ER Ca^2+^ leaky in the brain [16]. Based on these results, we speculated that calstabin2 might regulate spatial and emotional memory, at least in part, via modulating ER Ca^2+^ release from RyR2. Interestingly, it has been reported that, in cultured hippocampal and medium spiny neurons, ER Ca^2+^ release controlled by RyR is involved in the modulation of AMPA receptors (AMPARs) trafficking to the membrane [21]. Change in the abundance of AMPARs in the postsynaptic membrane is a major mechanism underlying various forms of synaptic plasticity, including LTP [22]. Thus, intracellular calcium leak via RyR2 might affect AMPARs trafficking, and then reduced LTP induction both in the hippocampus and LA.

Due to the lack of specific agonist and antagonist of RyR2, the role of calstabin2-RyR2 system in memory acquisition, consolidation and retrieval was not investigated. Several reports indicated that non-specific RyRs blocker dantrolene and agonist 4-chloro-m-cresol injected post-raining impaired and promoted long-term spatial memory, respectively [23–26]. Moreover, pre-training infusion of dantrolene also impaired long-term spatial memory [24, 26]. These reports suggest that RyRs regulate spatial memory during the acquisition and consolidation phases. However, whether RyRs regulate memory retrieval is still unknown.

## Data Availability

The data used in our study are available from the authors on reasonable request.

## Abbreviations

AAV: Adeno-associated virus
ACSF: Artificial cerebrospinal fluid
AD: Alzheimer disease
CS: Conditioned stimulus
ER: Endoplasmic reticulum
KD: Knockdown
KO: Knockout
LA: Lateral amygadala
LTP: Long-term potentiation
RyR: Ryanodine receptor
US: Unconditioned stimulus
